# Morphology and morphometry of adult nematodes on Sumatran elephants (*Elephas maximus sumatranus*) in Way Kambas National Park area, Indonesia

**DOI:** 10.14202/vetworld.2019.249-253

**Published:** 2019-02-13

**Authors:** Rahmania Prahardani, Lintang Winantya Firdausy, Yanuartono, Wisnu Nurcahyo

**Affiliations:** 1 Sains Veteriner Magister Program, Faculty of Veterinary Medicine, Universitas Gadjah Mada, Yogyakarta 55281, Indonesia; 2 Department of Internal Medicine, Faculty of Veterinary Medicine, Universitas Gadjah Mada, Yogyakarta 55281, Indonesia; 3 Department of Parasitology, Faculty of Veterinary Medicine, Universitas Gadjah Mada, Yogyakarta 55281, Indonesia

**Keywords:** morphology, morphometry, nematode, *Quilonia travancra*, Sumatran elephant

## Abstract

**Background and Aim:**

Worms from nematodes are the most numerous and the most detrimental in elephants. Most adult worms are located in the digestive tract. Nematode infection is at higher risk in young elephants, which caused several cases such as anemia, hypoalbuminemia, enteritis, and even death. This study aimed to determine the morphology and morphometry of adult nematodes on Sumatran elephants in Way Kambas National Park area.

**Materials and Methods:**

Nematode samples were obtained from Sumatran elephants’ feces (*Elephas maximus sumatranus*) in Way Kambas National Park, Lampung Province, after being given Kalbazen^®^ containing albendazole 1000 mg at a dose of 10 mg/kg by the veterinarian in charge of the National Park area. For the morphological and morphometric examinations, we used an Olympus BX 51 microscope equipped with Olympus DP 12 camera and were conducted at the Parasitology Laboratory, Faculty of Veterinary Medicine, Universitas Gadjah Mada. The scanning electron microscopic (SEM) analysis was carried out at the Biology Research Center of the Indonesian Institute of Sciences (Lembaga Ilmu Pengetahuan Indonesia).

**Results:**

The results of macroscopic observations of the obtained nematodes showed that the nematodes which were found have the characteristics of round, slim, and white color. The size of a female worm was larger than a male worm. Microscopic examination in four anterior papillae indicated that the dorsal lobe in the copulatory bursa was longer than lateral lobe. The result of inspection with the SEM showed a leaf crown consisting of 10 elements, a pair of amphids laterally, and two pairs of papilla in a submedian region.

**Conclusion:**

Based on our morphology and morphometry examinations of adult nematodes in Sumatran elephant (*E. maximus sumatranus*) in Way Kambas National Park area, the adult nematodes which were found are species of *Quilonia travancra*.

## Introduction

There is limited information regarding the identification of the taxonomy and life cycle of nematodes in an elephant; this is related to know the risks posed by worm infection and preventable prevention. Nematodes are the main cause of the most detrimental infection, especially in young elephants in which it may cause several cases such as anemia, hypoalbuminemia, and enteritis [1]. Stool examination in wild elephants in Sri Lanka shows 100% prevalence of the wild elephants infected by strongyle worms [2]. The highest prevalence of strongyle worm infection occurs in Bornean elephants in the Lower Kinabatangan Wildlife Sanctuary [3] and African elephants in Botswana [4]. At the examination of worm eggs in Asian elephant feces in Thailand, strongylids, ascharids, and trichurids types of egg were found [5], and at the examination of worm eggs in Asian elephants in India, strongyle and *Strongyloides* spp. type of eggs were often found [6].

Morphological identification is used to identify adult nematode; moreover, molecular identification mostly supports the result of morphological identification. Molecular identification has provided alternative approaches for the identification such as individual egg and worm of some nematode can be identified accurately to species. Identification of strongyle nematodes mainly depends on the morphology of adult male worms. The characteristics which are used to differentiate between species include the number of corona radiata in the anterior part, the shape and length of the esophagus, the shape and length of the dorsal lobe in the copulatory bursa, the location of the vulva, and the display of the tail in females [7].

This study aimed to determine the morphology and morphometry of adult nematodes on Sumatran elephants in the Way Kambas National Park area.

## Materials and Methods

### Ethical approval

This study has obtained permission from the Way Kambas National Park Office through a Conservation Area Entry License with number: SI. 484/BTNWK-1/2018, The Ministry of Environment and Forestry, General Directorate of Natural Resources Conservation and Ecosystems with SK number: 246/KSDAE/SET/KSA.2/6/2018, letter of recommendation for taking SATS-DN research samples from the Indonesian Institute of Sciences with number: B.3288/IPH.1/KS.02.04/X.2017, and Bengkulu Natural Resources Conservation Center with Number S.43/K.10/TU/PPN/06/2018.

### Tools and materials

The tools used for adult worms sampling were gloves, plastic trays, Petri dishes, glass tubes, and label paper. The tools used for worm morphology identification were object glass, cover glass, Olympus BX 51 microscope equipped with Olympus DP 12 digital camera, and scanning electron microscopic (SEM) JSM 5310 LV. The materials used in this study were adult nematodes, albendazole (Kalbazen®), aquades, lactophenol, and 70% alcohol.

### Parasites collection

Nematode worms were obtained from feces of Sumatran elephants in the Way Kambas National Park area after the anthelmintic administration in the form of Kalbazen^®^ 1000 mg at a dose of 10 mg/kg by veterinarians of the Way Kambas National Park. There were many worms found in the feces, but only 60 nematodes were suitable for this research. The prescription was done by measuring the weight of the elephant and counting the dose of drug administration based on the weight; then, the drug was put into a banana and given to the elephant by the mahout. Weight calculation was done by estimating body weight by measuring chest circumference and shoulder height according to Kurt [8]. The collected worms were immediately washed using aquadest and were put into a tube containing lactophenol for examination with a light microscope and 70% alcohol for morphological examination using an SEM.

### Morphological and morphometry identification

Morphological identification was performed by observing worms including mouth collar, papilla, buccal capsule, esophagus, vulva, anus, tail, speculum, and copulatory bursa using an Olympus BX51 Microscope equipped with Olympus DP Digital Camera 12. Worm measurements were using objective micrometer and objective ocular micrometer. Nematodes morphology was identified in morphological and morphometric character.

Identification of worm ultrastructures includes the anterior and posterior parts. The worm samples were fixed in 70% alcohol and then were sent to the Indonesian Institute of Sciences Biology Research Center. Worms were cleaned and soaked in cacodylate buffer for 2 h and agitated in an ultrasonic cleaner for 5 min. Each sample was put into 2.5% glutaraldehyde for 2-3 h and then were fixated in 2% tannic acid for 6 h. Then, they were washed with cacodylate buffer for 15 min. The washing process was repeated 4 times and the second washing process was carried out with 1% osmium tetroxide for 1 h and the third washing process was done with distilled water for 15 min. The dehydration steps were carried out by soaking the sample into multilevel alcohol from 50% alcohol for 5 min repeated 4 times, then soaked in 70%, 85%, and 95% alcohols for 20 min each, and soaked in absolute alcohol for 10 min twice at room temperature. The drying stage of the sample was done by immersing it in tertiary butanol for 10 min and repeated 2 times; then the samples were frozen in the freezer and dried with a freeze dryer. The dried samples were placed on a stub coated with gold (Au), and with an ion coater machine for 15 min and observed with the JSM 5310 LV SEM.

## Results

A total of 60 adult nematode worms consisted of 30 males and 30 females were taken and put into lactophenol solution (20 males and 20 females) to be examined morphologically using a light microscope, while 70% alcohol solution (10 males and 10 females) is used for the SEM examination.

### Morphology and morphometry description

**Figure-1:**
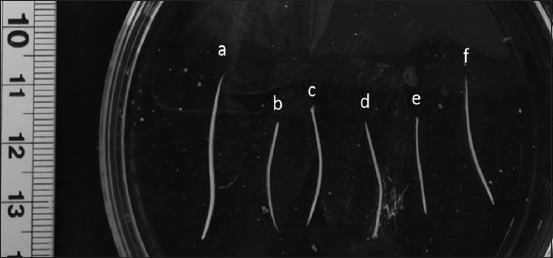
Adult nematodes in a Petri dish: (a), (b), (f) female nematode; (c), (d), (e) male nematode.

The results of macroscopic observations of the obtained nematodes showed that they have the characteristics of round, slim, and white color (Figure-1).

**Figure-2:**
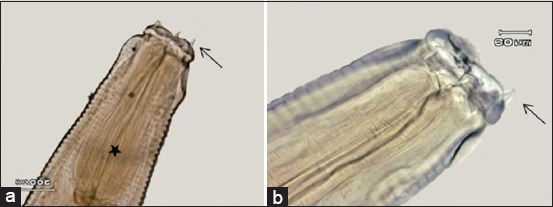
Anterior end of nematode (a) papillae (arrow), esophagus (asterisk); (b) papillae (arrow).

The male worms were 13-18 mm in body length with a maximum 0.51-0.66 mm in body width, while the female worms were longer in size, between 19 and 28 mm, with a maximum body width of 0.74-1.00 mm. The cuticular striation distance in male worms was 29.97-30.78 μm whereas in female worms was 34.83-39.69 μm. The diameter of the mouth collar in male worms was 0.17-0.18 mm while in female worms was 0.26-0.29 mm. Based on observations with a light microscope, there were four papillae that stood out at the anterior part (Figure-2). The length of the esophagus in male worms was 0.56-0.61 mm, while the length in female worms was 0.68-0.72 mm. The nerve ring in the male worm was located between 0.23 and 0.29 mm from the anterior body, while the female worm was located between 0.34 and 0.4 mm from the anterior body.

**Figure-3:**
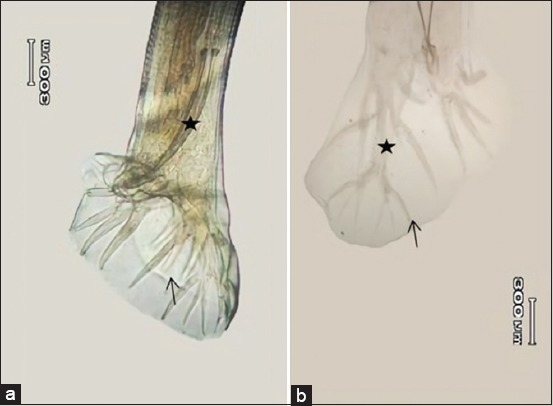
Posterior end of male nematode (a) bursa copulatrix of male (lateral view) external dorsal ray (arrow), spicules (asterisk). (b) Bursa copulatrix of male (dorsolateral view) dorsal ray (asterisk), rami (arrow).

Male worms typically featured with copulatory bursa at the posterior part of the body (Figure-3). The copulatory bursa was divided into three lobes, in which dorsal lobe was longer than the lateral lobe (Figure-3). The dorsal ray part in dorsal lobe was divided into two branches. Each of branch consisted of three rami which had the same length (Figure-3). The dorsal ray length was 0.73-0.87 mm. Externo-dorsal ray emerged from the main branch of dorsal ray (Figure-3). The length of spiculum was 0.75-0.92 mm.

**Figure-4:**
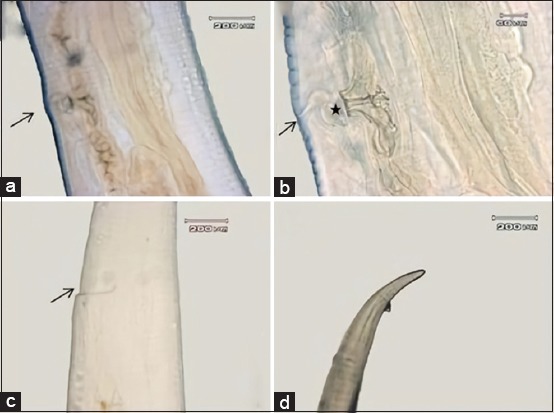
Posterior end of the female nematode. (a) Vulva (arrow). (b) Vulva (arrow), vagina (asterisk). (c) Anus. (d) Tail end.

The vulva of female worm located at one-third from the posterior part of the body. Shape of the vulva was slightly protruded from the body surface (Figure-4). The vulva located 4.7-5.87 mm from the tail end. The distance between vagina and vulva was about 0.08-0.09 mm. Shape of the anus was looked like a deep set which was concaving from vulva 2.79-3.59 mm toward posterior (Figure-4). The length of the female worm’s tail was about 1.9-2.43 mm with rather a dull end tail’s shape.

### SEM

**Figure-5:**
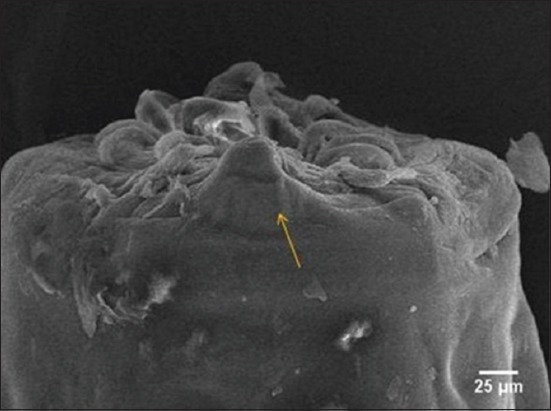
Anterior end of nematode. Amphid (arrow).

The result of the worm’s anterior part examination, specifically the mouth collar (Figure-5), indicated that there were two pairs of long and slim papilla at the submedian part and a pair of short or amphid papilla at the lateral part. Amphid, from the lateral view, looked like a mountain with rounded anterior part (Figure-5).

**Figure-6:**
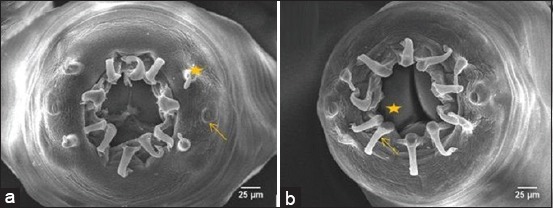
The end face view of adult nematode. (a) Four papillae (asterisk), amphid (arrow). (b) Mouth lobe (asterisk), leaf crown/corona radiata (arrow).

This worm had external leaf crown/corona radiata surrounding buccal capsule and did not protrude from the head surface, so it is only possible to be observed with SEM. External leaf crown consisted of 10 elements and bent to distal. There were three lobes of mouth which could be seen under buccal capsule (Figure-6).

**Figure-7:**
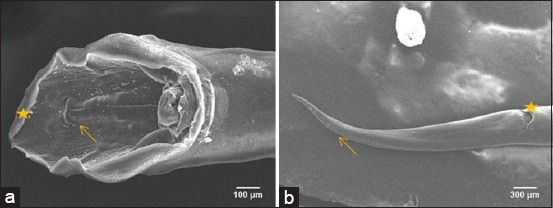
Posterior end of nematode. (a) Posterior end of male nematode. Dorsal lobe of bursa copulatrix (asterisk), dorsal ray (arrow). (b) Posterior end of female nematode. Anus (asterisk), tail (arrow).

Copulatory bursa’s dorsal lobe of male worm was longer than the lateral lobe, and its dorsal ray had two branches (Figure-7). Female worm’s anus part looked like a hollow space, and its tail end was slight dull (Figure-7).

Morphological and morphometric observations which had been carried out using a light microscope and SEM on adult nematodes originated from Sumatran elephants (*E. maximus sumatranus*) in the Way Kambas National Park area are from the species of *Quilonia travancra*.

## Discussion

Taxonomists use key identification to help distinguish species from adult nematodes morphologically.

Genus *Quilonia* belongs to the subfamily Cyathostominae and family Strongylidae. One of the characteristics of Cyathostominae is that there is external or internal corona radiata with several elements that emerge from the base of the buccal capsule. Genus *Quilonia* has a morphology in which the walls of the buccal capsule are not attached to the lining of the buccal cavity. The vulva is posterior to one-third of the body, and the vagina is very short. Dorsal ray in the copulatory bursa has two branches, and there are three rami in each branch. The genus of *Quilonia* is present in elephants or rhinos [9].

*Quilonia* has four prominent papillae in the submedian section which are used as a touching device on the worm and a pair of amphids in the lateral section of the mouth collision. Amphid is a pair of sensory glands located laterally at the head. Leaf crown consists of several elements with a slim shape. The dorsal lobe in the copulatory bursa of the male worm is longer than the lateral. The external dorsal ray comes from the main branches of the dorsal ray, and the dorsal ray is divided into two branches. Female worms have vulva located in posterior to one-third part of their body. The tail of the female worm is straight, long, and somewhat dull. *Quilonia* spp. is hospes in elephants and rhinos. *Quilonia renniei* resides in Indian, Burmese, or Indonesian elephants, besides that there is a species of *Q. travancra* which can occupy in Asian elephants (India or Burma) [10].

According to Baylis [11], to differentiate the species *Q. renniei* and *Q. travancra*, we can use the number of corona radiata/leaf crown and the length of the dorsal ray. *Q. renniei* has an external leaf crown consisting of 18 elements and protrudes from above the surface of the head, and the length of the dorsal ray is 0.35 mm. Species *Q. travancra* has an external leaf crown consisting of 10 elements and does not protrude above the surface of the head, and the dorsal ray length is longer than *Q. renniei* which is about 0.85 mm.

Male *Q. travancra* have been identified by Carreno *et al*. [12], which were taken from elephant feces after administering mebendazole anthelmintic. The worm has the main characteristic of 8-10 leaf crowns or corona radiata which are curved toward the distal direction.

Morphological observations were carried out using light microscopy and SEM on adult nematodes derived from Sumatran elephants (*E. maximus sumatranus*) in Way Kambas National Park area and were matched with morphological keys according to Anderson *et al*. [9] and Yamaguti [10] about the genus of *Quilonia*. Based on the morphological key according to Baylis [11] and research conducted by Carreno *et al*. [12], the nematode worm is a species of *Q. travancra*.

## Conclusion

Based on our morphology and morphometry examinations of adult nematodes in Sumatran elephant (*E. maximus sumatranus*) in Way Kambas National Park area, the adult nematodes which were found are species of *Q. travancra*.

## Authors’ Contributions

The research was determined, managed, and supervised by WN. RP and LWF took samples, recorded samples, and sample analysis. WN and RP arranged, analyzed, and wrote the report. Y worked overall observation of the experiment and the manuscript writing. All authors have read and approved the final manuscript.
